# Shared micromobility, perceived accessibility, and social capital

**DOI:** 10.1007/s11116-024-10521-5

**Published:** 2024-08-21

**Authors:** Zihao An, Caroline Mullen, Xiaodong Guan, Dick Ettema, Eva Heinen

**Affiliations:** 1 Institute for Transport Studies, University of Leeds, Leeds, UK; 2 Department of Land Economy, University of Cambridge, Cambridge, UK; 3 Cambridge Centre for Smart Infrastructure and Construction, University of Cambridge, Cambridge, UK; 4 Cambridge Centre for Environment, Energy and Natural Resource Governance, University of Cambridge, Cambridge, UK; 5 Department of Human Geography and Spatial Planning, Faculty of Geosciences, Utrecht University, Utrecht, The Netherlands; 6 Department of Transport Planning, Faculty of Spatial Planning, Technische Universität Dortmund, Dortmund, Germany; 7 Department of Architecture and Technology, Norwegian University of Science and Technology, Trondheim, Norway

**Keywords:** Shared micromobility, Social capital, Bike sharing, Shared e-scooter, Accessibility

## Abstract

While the impacts of shared micromobility (SMM) on the environment and transport systems are being extensively researched, its societal implications and the influence of the social environment on the use of SMM remain largely unexplored. In this research, we investigate the interrelationships between the use of SMM, perceived overall accessibility, and social capital. We focus on two types of SMM – shared bikes and shared e-scooters – in three European countries: the Netherlands, England, and Sweden. We measure perceived overall accessibility through a multicriteria subjective evaluation of individuals’ ability to reach regular destinations, services, and activities. We consider multidimensional social capital measures: social trust, cooperativeness, reciprocity, network bonding, and network bridging. We use multivariate models to investigate the associations between perceived overall accessibility, SMM use, and social capital, and examine the dominant direction of these associations using the direct linear non-Gaussian acyclic model (DirectLiNGAM) and direction dependence analysis (DDA). We find that lower levels of perceived overall accessibility may contribute to lower levels of social trust, reciprocity, and cooperativeness. However, individuals with a lower level of perceived overall accessibility tend to use shared bikes more frequently, which in turn, may increase their social trust and cooperativeness. We also find that increased shared e-scooter use may contribute to increased network bonding, yet the frequency of use has no relation with perceived overall accessibility. Our research suggests that the introduction of shared bikes alone, independent of other measures aimed at encouraging their use, may help mitigate individual differences in social capital. We argue that the applied DirectLiNGAM and DDA help gain deeper insights into the likely causal relationship between transport and social capital in non-intervention studies.

**Supplementary Information:**

The online version contains supplementary material available at 10.1007/s11116-024-10521-5.

## Introduction

Micromobility refers to lightweight vehicles that are typically operated below speeds of 25 km/h (Behrendt et al. 2022). Shared micromobility (SMM) has attracted growing attention in transport policymaking and the academic field, as it offers flexible transport alternatives for short-distance trips (Shaheen and Cohen 2021). While SMM’s impacts on the environment (Bozzi and Aguilera 2021; Zhang and Mi 2018) and transport systems (Wang et al. 2022; Liu and Miller 2022) are being widely researched, the societal implications of SMM, and how the social environment shapes the use of SMM, are relatively unexplored. In this research, we seek to contribute to these understudied topics by investigating the interrelationships between SMM use, social capital, and perceived overall accessibility.

Social capital describes norms and networks that arise from connections and relationships between and amongst individuals (Woolcock and Narayan 2000). The concept posits these norms and network features, such as social trust, reciprocity, cooperativeness, network bonding, and network bridging, enable individuals to act collectively for their mutual benefit (Coleman 1990; Fukuyama 1995; Putnam et al. 1993). Social capital is an important determinant of individuals’ well-being and quality of life (Kawachi et al. 2008; Villalonga-Olives et al. 2018), plays a vital role in the generation of social inclusion and exclusion (Stanley et al. 2010), and significantly contributes to advancing the development of society (Rupasingha et al. 2000). It is also suggested that social capital helps explain a range of behaviours, such as health, pro-environmental, and economic behaviours (e.g., a choice of transport mode) (Wan and Wencui 2022, Guzman et al. 2023; Nieminen et al. 2013). A nuanced understanding of the determinants of social capital, and how it impacts behavioural outcomes, is, therefore, crucial for fostering social and environmental sustainability, as well as for offering valuable insights into policy formulation and evaluation.

In the past two decades, the concept of social capital has emerged in the field of transport (Schwanen et al. 2015; Stanley et al. 2010). Transport may play an important role in facilitating the acquisition and accumulation of social capital through its influence on opportunities provided for social interactions and co-presence, i.e., the status of being with others (Currie and Stanley 2008). On the other hand, studies also posit that transport acts partially as a derived demand stemming from social interactions and, consequently could be influenced by social capital (Kim et al. 2018). Existing studies have predominately sought to investigate the transport-social capital relationship from the standpoint of mobility, which focuses on the potential that ease of moving has in shaping social capital (Preston and Rajé 2007). These studies have centred on the association between individuals’ social capital and the availability or use of transport options, with a particular emphasis on public transport, personal cars, and walking (Utsunomiya 2016; Boniface et al. 2015; Kamruzzaman et al. 2014).

Yet, existing studies on the transport-social capital relationship share three limitations. First, there is a scarcity of research focusing on emerging yet prevalent transport options, such as SMM. Second, limited empirical attention has been given to how accessibility, particularly perceived overall accessibility, which concerns the perceived ease of reaching various (interested) destinations, services, and activities (Pot et al. 2021), is connected with social capital. Gaining insights into the relationship between this construct and social capital, and how transport options, especially those emerging ones, are involved in such a relationship could have valuable implications for sustainable urban mobility planning and lead to the identification of further research questions. Third, it remains unclear whether transport acts primarily as an antecedent of social capital, or vice versa. This poses challenges for developing effective transport policies in the promotion of social capital and for understanding the determinants of transport.

This research aims to investigate the interrelationship between the use of SMM, perceived overall accessibility, and social capital. We focus on two types of SMM, i.e., shared bikes and shared e-scooters, in three European countries: the Netherlands, England, and Sweden. Our research contributes to the existing body of literature on SMM and on the social capital-transport relationship through three key aspects. First, it offers novel insights into the impact of SMM on social capital. Second, it provides evidence of the relationship between perceived overall accessibility and multidimensional social capital. Third, it applies innovative statistical approaches to explore the (dominant) directions in the association between social capital and the use of SMM, which facilitates an in-depth understanding of the likely causal relationship between social capital and transport.

## Background

### Concept of social capital

Social capital is a polysemic concept that has evolved over time, and there is no unanimous agreement as to the precise definition and components of the concept (Bjørnskov and Sønderskov 2013). One of the earliest systematic expositions of the concept came from Bourdieu (1986), who presented social capital as a property of individuals, and defined the concept as ‘the aggregate of the actual or potential resources which are linked to possession of a durable network of more or less institutionalised relationships of mutual acquaintance and recognition’ (p. 248), taking an exclusive network perspective into account.

In contrast, several other scholars have sought to conceptualise social capital within a larger sphere, recognising its relevance with broader social structures, as well as more comprehensive sociological processes (Coleman 1990; Fukuyama 1995; Putnam et al. 1993). For example, Coleman (1990) viewed social capital as an aspect of a social structure, and defined the concept by its function in facilitating individuals’ actions within a social structure. He proposed that social capital encompasses three broad categories: (1) obligations and expectations, such as trust, which are built upon shared understanding within a structure; (2) information channels, such as network ties, which underscore the importance of communication and knowledge-sharing within a structure; and (3) norms and sanctions, such as norms of cooperativeness and reciprocity, which facilitate cohesion within a structure. Coleman’s (1990) formulation has inspired subsequent research in the field, notably including the influential contributions of Putnam et al. (1993).

In line with Coleman’s (1990) conceptualisation, Putnam et al. (1993) also treated social capital as a public good, which is formed within social structures. Putnam et al. (1993) defined social capital as ‘features of social organizations, such as networks, norms and trust that facilitate action and cooperation for mutual benefit’ (p. 167). Putnam (1995) also highlighted that civic virtue is embedded in social capital, and therefore the level of civic participation is indicative of that of social capital. Putnam (2000) further developed the concept of social capital to include bonding capital (i.e., social resources that lie within one’s close network) and bridging capital (i.e., social resources that extend outside of one’s immediate network). A key feature of Putnam’s (1995, 2000) conceptualisation is that it is macro-oriented, which primarily focuses on social capital’s implications at the aggregate level (e.g., the community level) (Grossman 2013). Despite this, analyses based on this conceptualisation have been widely applied at the micro level (e.g., individual level) in existing studies (Kamruzzaman et al. 2014; Snelgrove et al. 2009).

In summary, these diverse perspectives suggest that social capital comprises multiple core components, such as trust, reciprocity, cooperativeness, civic engagement, and participation in social networks, which work together to facilitate resource acquisition, cooperative action, and mutual benefit.

### Transport and social capital

Currie and Stanley (2008) outlined the theoretical linkages between transport and social capital, suggesting that the opportunities for social interactions and co-presence, which are either created or hindered by transport systems, play a significant role in shaping this relationship. Studies have highlighted the importance of overcoming spatial constraints for social interactions (Axhausen 2005; Hill and Dunbar 2003). Evidence has also suggested that increased time spent travelling reduces people’s willingness and frequency to socialise (Besser et al. 2008). Frequent social interactions are crucial for the establishment, maintenance, and strengthening of social relationships, thus contributing to stable and diverse social networks (McPherson et al. 2001). These interactions could also form the basis for increased levels of social trust, i.e., generalised trust in other people including both familiar and unfamiliar, local and non-local (Glanville et al. 2013), and cooperation (Duffy and Ochs 2009).

Transport plays a vital role in facilitating co-presence. For example, when using public transport like buses, unplanned co-presence (e.g., casual encounters on streets) may occur from walking to the bus station, waiting for the bus, to being on the bus, and reaching the intended destination. In contrast, car use limits unplanned co-presence (Putnam 2000). Co-presence is essential for building social capital, particularly in today’s digital age, as it augments opportunities for face-to-face contact (Nilsson and Mattes 2015; Rocco 1998); such contact, enriched by facial expressions, body language, and eye contact, can deepen interpersonal connections (Currie and Stanley 2008). This holds not only for planned co-presence – as it is a crucial basis for fulfilling a variety of obligations (Urry 2002) – but may also extend to unplanned co-presence. Unplanned co-presence in urban spaces may encourage acquaintanceship ties (Wickes et al. 2019), thereby promoting the development of shared norms and social trust (Bradley 2015; Jacobs 2016). However, it is noteworthy that some instances of co-presence are not pleasant, which can negatively affect individual perceptions (Löfgren 2015), and consequently, may not facilitate social capital. Moreover, the role of co-presence in facilitating social capital may vary depending on certain characteristics, such as the duration and quality of co-presence (Nilsson and Mattes 2015).

The emotional implications of travel have also been suggested as a factor linking transport and social capital (Mattisson et al. 2015). The choice of modes and the distance travelled lead to different degrees of flexibility, predictability, and satisfaction (An et al. 2021, 2022; De Vos et al. 2016; Ettema et al. 2012). Travel disturbances, compounded by overall dissatisfaction, can exacerbate feelings of hostility and stress (Koslowsky et al. 1995). This heightened emotional distress tends to decrease the willingness for social interaction, consequently undermining social participation and trust (Mattisson et al. 2015).

Conversely, the existing literature also posits that individuals’ social capital potentially exerts influence over their travel behaviour. Establishing and maintaining social networks is accompanied by the necessity of participating in outdoor activities. The size, diversity, and spatial distribution of social networks are associated with the frequency, types, and locations of these activities, which in turn affect individuals’ transport choices (see, e.g., Kim et al. (2018) for the review). Beyond social networks, research also suggests that social capital in other domains may have an impact on travel mode choices (Guzman et al. 2023; Di Ciommo et al. 2014). For example, Guzman et al. (2023) found that higher levels of social trust and cooperativeness were linked with higher willingness to use a new public transport alternative (i.e., a cable car service), potentially attributed to the positive association between these social capital variables and the sense of community.

Empirical studies have been conducted to investigate the association between transport and social capital, based on multivariate linear models and cross-sectional data. Stanley et al. (2010), in a study conducted in Australia, found an increase in trip frequency is associated with more frequent interaction within bridging networks (networks beyond the immediate social circle). More studies on this topic have centred on how social capital correlates with the use and availability of various transport options. Mattisson et al. (2015) found that in Sweden, car commuters, compared to those using active modes, demonstrated lower levels of social trust and participation. Commuting by public transport, however, did not yield a significant difference in these types of social capital compared to car and active commuting. Aggregate-level evidence also supports a negative correlation between car use and social capital. Rahn et al. (2009) indicated that US communities with a higher percentage of individuals commuting by car for more than 45 min tended to experience a lower level of social trust. Similarly, Utsunomiya (2016) observed that Japanese municipalities with more private cars per capita showed reduced levels of social trust and participation. Conversely, municipalities logging more bus kilometres per capita correlated with higher levels of social participation and social network capital, a point echoed by Qin and Fukuda (2023). Using a composite index of social capital, Qin and Fukuda (2023) revealed that Japanese municipalities with a greater proportion of bus or train commuters correlated with higher area-level social capital.

Studies have provided indirect evidence on the transport-social capital relationship by investigating how the residential built environment correlates with social capital. It has been found that transit-oriented development communities, characterised by ready accessibility to public transport facilities, display increased levels of social trust, reciprocity, neighbour connectedness, and social participation (Noland et al. 2016; Kamruzzaman et al. 2014; Utsunomiya 2016) than other areas. Individuals in walkable neighbourhoods, characterised by easy access to facilities within walking distance, high land use mix, and efficient street network connectivity, are prone to be acquainted with their neighbours, engage in political participation, trust others, and be socially active (Leyden 2003; Rogers et al. 2013; Du Toit et al. 2007).

The literature on the built environment-social capital relationship also suggests the role of accessibility in supporting social capital building. This resonates with research on transport poverty and transport-related exclusion (Schwanen et al. 2015; Lucas 2012; Preston and Rajé 2007). The latter emphasises the simultaneous consideration of both mobility and accessibility dimensions in a holistic framework for understanding social exclusion. For example, low-income families lacking car access might exhibit limited mobility, yet maintain robust accessibility if regular activities as well as local amenities and services are within walking distance. However, when examining accessibility’s influence on social capital, empirical studies have mostly applied spatial elements as proxies for measuring accessibility, and linked them with social capital. Yet, these proxies do not accurately reflect accessibility to potential destinations and activities as they are individually perceived, given that individuals differ in their environmental awareness, spatial knowledge, and transport conditions (Pot et al. 2021). The studies also predominately narrow their focus on accessibility to particular facilities (e.g., retail or parks) supported by specific transport options (e.g., public transport or walking). Such an approach fails to consider the vast spectrum of human activities and available transport alternatives.

The notion of perceived overall accessibility presents an opportunity to overcome the aforementioned limitations. It pertains to an individual’s subjective evaluation of how easily they can reach diverse regular destinations, services, and activities (Pot et al. 2021). Perceived overall accessibility diverges from the so-called ‘objective’ spatial elements-determined accessibility, being influenced by personal experiences, cognitive maps, and socio-economic factors (Pot et al. 2021; Mondschein et al. 2010). It encompasses not only spatial access but also various other factors, such as individual capability, assurance, and psychological comfort in accessing daily activities (Lättman et al. 2018; Wang et al. 2021; Koopmans et al. 2013). Recognising this differentiation is pivotal in comprehending the intricate relationship between accessibility and social capital.

Our review suggests that increased car use may be associated with lower levels of social capital, whilst the use of public transport and walking as well as residence in areas that are conducive to these options may correlate with higher levels of social capital. However, limitations exist in existing studies. First, there is a scarcity of research focusing beyond conventional transport modes, such as cars, public transport, and walking. Second, while transport is conceptualised as a cause of social capital in these studies, the dominant direction of this relationship remains unclear due to limitations associated with multivariate regression models in combination with cross-sectional research designs. Third, there is still a dearth of direct evidence examining the relationship between an individual’s perceived overall accessibility and social capital, and how using transport modes interacts with these two constructs within this relationship.

### Perceived overall accessibility, social capital, and SMM use

This subsection delineates the intricate associations to be examined in our research, specifically focusing on the interrelationships between perceived overall accessibility, social capital, and SMM use. We highlight that we aim to explore the ‘net impact’ of these elements on each other, *independent of* confounding factors such as individuals’ demographic and socioeconomic characteristics as well as residential locations. This focus facilitates a nuanced understanding of the interrelationship between these elements, particularly in the context of the Global North where these confounding variables tend to overlap. For example, existing research highlights the uneven distribution of SMM services, which are predominantly deployed in denser areas for profit reasons (Bai et al. 2021; Ricci 2015; Mooney et al. 2019),–areas that also correlate with a higher level of residents’ perceived accessibility (Walton et al. 2008). Moreover, studies show that shared bike users tend to be individuals who are educated, white, male, aged in their 20s or 30s, and with incomes above the average (Teixeira et al. 2021, Sophia et al.); e-scooter users are generally males and younger adults (Christoforou et al. 2021; Mouratidis 2022). While specific characteristics of shared e-scooter users remain largely inconclusive across different study locations, it has been shown that these users are associated with a medium-to-high household income in Malmö (Sales et al. 2021), a case study area in our research. Evidence on factors associated with higher levels of social capital in the Global North–namely being male, belonging to dominant ethnic groups, and having a higher socioeconomic status (Marsden 1988; Kawachi et al. 1997; van Tubergen and Volker 2014)–partially align with these revealed SMM user traits and advantages in perceived accessibility to various facilities and services, as extensively documented in the existing literature (Yasumoto et al. 2021; Field and Briggs 2001).

#### Perceived overall accessibility and social capital

Individuals perceiving overall accessibility disadvantages may encounter difficulties in acquiring and sustaining social capital, possibly as a consequence of reduced opportunities for social interaction and co-presence. Low levels of accessibility to facilities, particularly recreational and social facilities, limit the occurrence and frequency of social interaction (Sharmeen et al. 2014; Bennet et al. 2012). Studies also highlight the role of accessibility in shaping co-presence in specific locations. For example, it has been found that more co-presence tends to occur on streets with greater centrality in the networks–those more readily accessible to other locations–and public spaces that are linked by those streets (Maciel and Zampieri 2021; Peponis et al. 1997; Shen et al. 2019). Low-level perceived overall accessibility, therefore, might serve as a barrier to co-presence.

Conversely, the enhancement of social capital may also contribute to the improvement of individuals’ perceived overall accessibility. Social capital, such as social trust and reciprocity, constitute crucial components underpinning the sense of community (Yetim and Yetim 2014; Wu et al. 2019). A stronger sense of community correlates with higher perceived safety in neighbourhood environments (Lund 2002), and could translate to better local knowledge sharing, which may help individuals discover more efficient routes and more affordable transport options (Ergün and Avcı 2018). These elements potentially increase individuals’ perceived overall accessibility.

#### SMM use and perceived overall accessibility

Empirical evidence reveals that more SMM use tends to enhance individuals’ daily travel accessibility by providing flexible transport alternatives to short-distance trips and being efficiently integrated into public transport systems (Wang et al. 2022, Liu and Miller 2022, Smith and Schwieterman 2018, Qian and Niemeier 2019, Buehler et al. 2021, Filipe Teixeira et al. 2022). Increased use of SMM may therefore support a higher level of perceived overall accessibility.

In contrast, individuals perceiving overall accessibility disadvantages may also tend to use SMM more frequently. SMM users generally hold the belief that SMM facilitates easier access to regular destinations–even if such beliefs may not be correct, which motivates them to use SMM (Chen et al. 2022a; Fishman et al. 2014; Shaheen et al. 2012). The perceived benefits of SMM in accessibility promotion may increase its usage utility amongst individuals with perceived accessibility disadvantages. This, in turn, potentially leads to *adaptive behaviour* by using SMM more frequently, holding constant other correlates of SMM use and perceived overall accessibility, e.g., socioeconomic characteristics, residential contexts, and the availability of SMM, fixed. In contrast, those already satisfied with their existing accessibility may not be equally motivated to use SMM, despite its perceived benefits.

#### SMM use and social capital

The potential mechanisms by which the use of SMM facilitates the development of social capital are multifold. First, more SMM use enhances individuals’ accessibility to destinations and activities (Chen et al. 2022a, 2022b; Bai et al. 2021). For example, Chen et al. (2022a) illustrated how shared bike usage can facilitate easier access to a wide range of activities within subsistence, maintenance, and leisure domains. This fosters the establishment of social networks and greater social interactions. Second, higher use of SMM may lead to increased co-presence with individuals, both familiar and unfamiliar, local and non-local, thereby nurturing social capital. SMM use often necessities walking trips to SMM infrastructures (e.g., parking facilities) (Kabra et al. 2020). During these trips and around the infrastructures, particularly those located in high-traffic public spaces, spontaneous encounters and interactions may occur, potentially facilitating shared norms and a sense of community. Third, the satisfaction derived from the use of SMM may contribute positively to the formation of social capital. The convenience, flexibility, and efficiency offered by SMM may elevate the user’s overall travel satisfaction, when SMM is involved in trips or used as the primary mode (Chen et al. 2022b). This heightened travel satisfaction potentially makes individuals more receptive and active to social interactions (Mattisson et al. 2015). Fourth, studies suggest that value co-creation behaviour (e.g., reporting broken bikes and ensuring the convenience of bike access) along with the use of SMM helps establish sharing norms, trust and reciprocity (Lan et al. 2017).

In contrast, higher levels of social capital may also be a cause of increased use of SMM may also be, although direct evidence is scant. For example, SMM serves dual roles: as leisure activities (e.g., leisurely rides with friends, as reported by Chen and Chancellor (2020)) and as a transport option, both potentially aiding in the sustenance of social networks.

## Research design

### Study area and data

Our research focused on shared bikes and shared e-scooters in three European areas: Greater Manchester in England, Utrecht in the Netherlands, and Malmö in Sweden. These areas differ significantly in the availability of SMM services and individuals’ levels of cycling. *Greater Manchester* is a metropolitan county comprising ten cities and towns. In this region, cycling constitutes only 5% of total trips and cars dominate with a 52% share (Deloitte 2020). In Greater Manchester, at the time of data collection, an expanding public cycle-hire scheme existed in Manchester City, and a shared e-scooter trial was in place in Salford and Rochdale (Manchester City Council 2022; Kane 2022). Our research focused on Manchester City and Salford, where SMM was operated, along with their adjacent area, Trafford.

*Utrecht*, the third-largest city in the Netherlands, is known for its flat terrain and well-established cycling culture. 28% of all trips starting or ending in Utrecht are made by bikes, rising to 48.5% within the city (CBS 2020). SMM is less widespread in this city compared to other countries. There existed traditional bike-sharing programs, which were operated by the Dutch Railways. Newer services such as shared e-bikes, shared (e-)cargo bikes, and shared mopeds have been introduced recently. Notably, e-scooters are not available in Utrecht in a shared form.

*Malmö*, Sweden’s third-largest city, offers a favourable environment for micromobility due to its flat topography and commitment to sustainable urban planning. Approximately 26% of residents primarily use bikes for transport (Skåne 2022). The city provides a range of SMM options, including shared bikes, e-scooters, and e-bikes, which are operated by both municipal and private entities.

We collected data through an online questionnaire survey from July to September 2022. Utilising commercial panel services, we targeted a general population aged 18 and above in the studied areas, which helps ensure a diverse sample of both SMM users and non-users. The questionnaire, segmented into four sections, covers topics concerning demographics and socioeconomics, the usage patterns of SMM and other transport modes, attitudes towards SMM, and social capital. The survey was available in the local languages of each study area, with an English option, and it took an average of 22 min to complete.

We received 2056 completed survey responses, with a breakdown of 26% English, 22% Dutch, and 52% Swedish participants (Appendix A). The gender composition consists of 45% males and 55% females, and the average age of respondents is 41 years. 42% report utilising either shared bikes or shared e-scooters at least once in the year preceding the survey. 58% possess higher education qualifications, 28% hold upper secondary education, 7% attained secondary education, and 7% obtained other educational backgrounds. We asked respondents about their household income according to the officially reported household income quintile in their respective areas; 12%, 15%, 20%, 19%, and 20% of the analysed individuals were from households with income falling into the first through fifth quintiles (‘prefer not to say’ accounted for 15%), respectively.

Our sample displays certain variations in representativeness across subpopulations when broken down by specific areas. A critical reflection on this issue and its implications on the generalisability of our findings will be presented in SubSect. “Limitations and future directions”. Compared to area-level statistics, our sample exhibits an overrepresentation of females and native-born individuals across the studied areas^1^ (CBS 2023, Neilsberg 2024, ONS 2024, City Population 2023a, 2023b, 2023c). While our sample maintains a relatively even age distribution, it notably overrepresents younger age groups (17–34 years) in both Greater Manchester and Utrecht (ONS 2024; CBS 2023), relative to area statistics. In contrast, the Malmö sample slightly overrepresents all age groups except those aged 65 and over^2^ (Urbistat 2023). The Greater Manchester and Utrecht samples demonstrate a slight underrepresentation of individuals whose household incomes fall into the lowest and highest quintiles of their respective areas; in contrast, the Malmö sample overrepresents individuals from the highest income quintile while underrepresenting those from the lowest. In comparison to national statistics (OECD 2024), the sample from Greater Manchester exhibits a higher proportion of individuals with higher education qualifications, whilst the samples from Utrecht and Malmö are aligned with national levels.^3^

### Social capital measurements

We measured five types of social capital: (1) social trust, (2) cooperativeness, (3) reciprocity, (4) network bonding, and (5) network bridging, considering the multidimensional nature of the concept.

Social trust is measured by asking the respondents to answer a question, following the 2018 European Social Survey (ESS) (European Social Survey 2018): ‘Generally speaking, would you say that most people can be trusted, or that you can’t be too careful in dealing with people?’ Cooperativeness was determined through a question, following the 2018 ESS (European Social Survey 2018): ‘Would you say that most of the time people try to be helpful or that they are mostly looking out for themselves?’ Reciprocity was measured based on the respondents’ evaluations of a statement, following Socio-Economic Panel Group (2015): ‘I am ready to undergo personal costs to help somebody who helped me before.’ Social trust, cooperativeness, and reciprocity were all measured using a 7-point Likert scale that ranged from 1 ‘Strongly Disagree’, through 4 ‘Neither Agree nor Disagree’, to 7 ‘Strongly Agree’.

Network bonding and network bridging describe two distinct types of social networks, through which social capital can be produced (Stone 2003). Following the existing literature (Stanley et al. 2010; Perry et al. 2021), we measured network bonding based on the extent to which an individual interacts with (1) close and extended family; (2) friends and intimates; and (3) neighbours. Network bridging was measured based on the extent to which an individual interacts with (1) work colleagues and classmates; and (2) people associated with groups in their community groups (e.g. church, sporting, clubs, school, self-help or voluntary groups). For three measures of network bonding and two of the network bridging, the interaction frequency was measured using a seven-point scale: (1) (Almost) never; (2) 1–5 days a year; (3) 6–11 days a year; (4) 1–3 days a month; (5) 1–3 days a week; (6) 4–6 days a week; (7) Daily. We then calculated the sum of the scores across these categories to measure an individual’s network bonding and network bridging.

### Perceived overall accessibility measurements

We measured individuals’ perceived overall accessibility based on respondents’ evaluations on nine statements, using a five-point Likert scale that ranged from -2 ‘Strongly Disagree’ to + 2 ‘Strongly Agree’. These statements were determined based on three criteria for high-level perceived accessibility, as identified in the existing literature, and were construed following the MOBIMON study (Ettema et al. 2022). Specifically, high-level perceived overall accessibility for an individual when they perceive interested destinations and activities can be reached (1) in an ideal travel time (Preston and Rajé 2007; Wang et al. 2021);(2) at affordable costs (Church et al. 2000; Koopmans et al. 2013); (3) with a positive travel experience (Lättman et al. 2018; Jamei et al. 2022).

For criterion (1), we asked respondents to evaluate six statements related to their ability to reach regular destinations and activities within their ideal time: ‘I can easily reach all my regular destinations and activities’ and ‘I can easily reach [my workplace (or place of education)/the supermarket or local shopping areas/healthcare facilities/friends or relatives at their home/my gym, team, place of worship, or bobby clubs] in my ideal travel time’. For criterion (2), the respondents were asked to evaluate the statement on their travel costs: ‘I do not have to spend more money on necessary travel in a week than I can afford’. For criterion (3), the respondents were asked to evaluate two statements concerning their travel experience: ‘I feel safe while travelling to my regular destinations and activities’ and ‘I can usually travel in a way that is suited to my physical condition and abilities’. There was a high level of internal consistency between individuals’ evaluations of these nine statements (Cronbach’s alpha = 0.879), suggesting that individuals’ evaluations of these statements are suitable for reflecting the same construct. As such, we calculated the perceived overall accessibility indicator using sum scoring across these nine statements.

### SMM use measurements

The respondent was asked to report their frequency of using shared bikes and shared e-scooters in the 12 months leading up to our survey. We used a seven-point scale to record SMM use frequency: (1) (Almost) never; (2) 1–5 days a year; (3) 6–11 days a year; (4) 1–3 days a month; (5) 1–3 days a week; (6) 4–6 days a week; (7) Daily.

### Analytical approaches

#### Model specifications

We examined three sets of associations, each set involving several linear regression models: (1) perceived overall accessibility and social capital; (2) perceived overall accessibility and SMM use frequency; and (3) SMM use frequency and social capital. Table 1 shows the specification of models used to examine these associations. We controlled each model for a set of variables revealed to correlate with social capital (Kamruzzaman et al. 2014; Kaasa and Parts 2008), use of SMM (Younes et al. 2020; Blazanin et al. 2022), and perceived accessibility (Liu et al. 2021), to mitigate potential endogeneity. These controlled variables included individuals’ demographic and socioeconomic characteristics – i.e., age, gender, educational attainment, city/area of residence, country of origin, living alone or not, living with children or not, household income, employment status, and health conditions (Appendix A) – as well as the use and access of transport options, i.e., access to cars, and personal bikes, ownership of other types of micromobility (i.e., e-bikes, e-scooters, mopeds, e-skateboard), and the frequency of walking and using cars, local public transport, regional public transport, personal bikes, and personal e-bikes (Appendix B). In addition to these variables, each model was also controlled by fixed effects of residential location (i.e., postcodes) to (partially) account for the availability of SMM and residential contexts. We also included SMM use frequency in models (1) and perceived overall accessibility in models (2) as controlled variables, given their potentially close linkage, to ensure the investigated associations are not confounded by these variables.

**Table 1:** The associations examined

Examined Associations
Social Capital (DV) & Perceived Overall Accessibility (IV)
Shared Bike (DV) + Shared E-scooter Use Frequency (DV) & Perceived Overall Accessibility (IV)
Social Capital (DV) & Shared Bike Use Frequency (IV) + Shared E-scooter Use Frequency (IV)

*DV* Dependent variable; *IV* Independent variable

It is worth noting that, while the dominant direction between each pair of our focused variables–perceived overall accessibility, social capital, and SMM use–is unclear, we only examined each of the focused associations with only one variable as the dependent variable. This approach was adopted because reversing the dependent and independent variables does not alter our statistical inferences regarding the associations (i.e., *p* value and the sign of the associations would be the same) under an identical set of controlled variables.

Shared e-scooters were only available in Greater Manchester and Malmö at the time of data collection. Therefore, for models (2) and (3), where shared e-scooter use frequency was treated as a variable of interest, we also used samples of only individuals living in Greater Manchester and Malmö for sensitivity analyses to ensure the robustness of our results, even though all models were controlled for city/area of residence. Given the large number of variables considered, we tested the multicollinearity of the models using the variance inflation factor (VIF) and found no problematic multicollinearity arising from our variables of interest that could bias the interpretations of our results.

#### Dominant direction examinations

While cross-sectional regression models are useful in indicating the strength and robustness of associations between variables of interest, they provide limited insights into the dominant direction of the associations in non-intervention studies (Angrist and Pischke 2009). In our research, for example, a positive association between social capital and SMM use frequency could suggest that more frequent SMM usage may enhance social capital. Alternatively, it could also signify that a higher level of social capital leads to increased SMM use. To investigate the dominant direction of the significant associations identified in our multivariate analyses, we used two methods, namely, the direct linear non-Gaussian acyclic model (DirectLiNGAM) (Shimizu et al. 2011) and direction dependence analysis (DDA) (Wiedermann and von Eye 2015). These two methods are built upon different theories to detect dominant directions: DirectLiNGAM is developed based on direct acyclic graphs (DAGs) and residuals’ non-Gaussianity and independence, whereas DDA relies on asymmetric properties of outcomes and predictors. Despite widespread usage of both methods in existing literature (Itahashi et al. 2020; García-Velázquez et al. 2019; Akkuş and Peker 2022, Dierckx et al. 2022), no studies have compared their statistical performance. We therefore applied both methods to ensure the robustness of our results.

DirectLiNGAM functions as a (likely) causal discovery algorithm. This algorithm assumes that the structure of the interested data follows a linear non-Gaussian acyclic model (LiNGAM), meaning the data are assumed to be generated from a linear process that can be represented by a DAG, and that the noise in the data generation process is assumed to follow independent and identically distributed (i.i.d.) non-Gaussian distributions. To ascertain the dominant directions of causality, DirectLiNGAM first executes a series of least squares regressions between a target variable (as an outcome) and all other variables (as predictors) to extract a set of residual vectors. Second, it assesses the pairwise independence between the target variable and each residual vector using a pairwise likelihood estimation (Hyvärinen and Smith 2013). Based on this analysis, the algorithm identifies the most probable exogenous variable and establishes pairwise causal orders. This identification is grounded in the Darmois-Skitovitch theorem (see Lemma 1 from Shimizu et al. (2011)), which shows that a variable’s exogeneity in a LiNGAM framework is confirmed if it is independent of the extracted residual vectors. Third, once an exogenous variable is identified, DirectLiNGAM removes it from the dataset. It can be proved that the remaining variables still conform to a LiNGAM (see Lemma 2 from Shimizu et al. (2011)), allowing the process to iteratively continue identifying and removing further exogenous variables, thereby simplifying the likely causal structure. These three steps are repeated until all the pairwise causal orders are determined. After this process, paths between variables whose weights are insignificantly different from zero, as determined by the Wald test, are removed from the DAG. A variable is considered to be a cause of another variable when it has a lower causal order in the sequence and possesses direct or indirect paths to the latter. We refer our readers to Shimizu et al. (2011) for more details on DirectLiNGAM.

DDA, on the other hand, utilises high-order moments to identify the dominant direction. Dodge and Rousson (2000) and Dodge and Yadegari (2010) demonstrated that the Pearson correlation exhibits asymmetric properties when considering the third and fourth moments: $r_{xy}^{3} = {{s_{x} } \mathord{\left/ {\vphantom {{s_{x} } {s_{y} }}} \right. \kern-0pt} {s_{y} }}$ and $r_{xy}^{4} = {{\kappa_{x} } \mathord{\left/ {\vphantom {{\kappa_{x} } {\kappa_{y} }}} \right. \kern-0pt} {\kappa_{y} }}$, where *x* and *y* are respectively the predictor and outcome for a linear data generation process; *r*_*xy*_ is the Pearson correlation between *x* and *y*; *s* and *κ* respectively denote skewness and kurtosis. DDA asserts that the difference in the absolute value of skewness (Δ*s*) and kurtosis (Δ*κ*) of a pair of covariate-adjusted variables can be used to determine the dominant direction, as *r*_*xy*_ is bounded between −1 and + 1: the absolute values of *s* and *κ* of the covariate-adjusted outcome will always be smaller than the those of the covariate-adjusted predictor (Dodge and Yadegari 2010). Therefore, DDA essentially identifies the dominant direction by assessing which covariate-adjusted dependent variable, from either the target or alternative model, conforms more closely to a normal distribution. If Δ*s* and/or Δ*κ* between the target and alternative models concerning the outcome are negative, and there is no significant discrepancy between the signs of Δ*s* and Δ*κ* (e.g., a discrepancy exists if Δ*s* is significantly larger than 0, whilst Δ*κ* is significantly smaller than 0), the target model is suggested to fit the data generation process better. However, in cases where a discrepancy between these signs exists, DDA is unable to determine the dominant direction (Pornprasertmanit and Little 2012). DDA’s methodology is further extended to residual distribution since the nonnormality of a misspecified predictor can be preserved in the error term (Wiedermann and von Eye 2015). Consequently, residuals from a model that more accurately mirrors the data generation process will exhibit a distribution that is closer to normal. In our research, the target models were established as per the models in Table 1. We calculated *s* and *κ* of each pair of target and alternative models on the outcome and residual distributions, and determined the confidence interval of Δ*s* and Δ*κ* using bootstrapping with 10,000 iterations.

The presence of outliers can compromise the robustness of the inference of DirectLiNGAM and DDA (Cao et al. 2022; Wiedermann et al. 2020; Leyder et al. 2023). To address this issue, we removed outliers prior to examining the dominant directions through the use of median absolute deviation (MAD), a robust outlier detection metric (Pornprasertmanit and Little 2012). Outliers were identified when residuals deviated more than three times the MAD from the median of residuals in either the target or alternative models (Leys et al. 2013).

## Results

### Descriptive analyses

In the 12 months leading up to our survey, 31% of individuals used shared bikes at least once. 19% reported a usage frequency of shared bikes at least 1–3 days monthly, and the daily users were minimal at 4%. Significant (*p* < 0.001, Pearson’s chi-squared test) variations in shared bike use existed across the studied areas. Utrecht reported the highest user rate (44%), followed by Greater Manchester (42%) and Malmö (21%). Regarding shared e-scooter use, 25% of the entire sample, and 32% of the sub-sample from Greater Manchester and Malmö, where shared e-scooters are available, had utilised these at least once. 14% reported using shared e-scooters for a minimum of 1–3 days per month, whilst the percentage of daily users was less than 2%. Greater Manchester significantly (p < 0.01) had a larger share of shared e-scooter users than Malmö (37% vs. 30%).

On average, social trust, cooperativeness, reciprocity, network bonding, and network bridging respectively scored 4.3 (S.E. = 1.5; median = 5), 4.2 (S.E. = 1.4; median = 4), 5.1 (S.E. = 1.5; median = 5), 15.8 (S.E. = 3.3; median = 16), 7.6 (S.E. = 3.3; median = 8). This suggests that individuals on average display a neutral disposition (neutral point = 4) towards social trust and cooperativeness, but exhibit a mildly positive disposition towards reciprocity. For network-based social capital, the average frequency of interacting with members in their close social network is approximately 1–3 days a week (see, the measurement details in subSect. “SMM use measurements”). In contrast, interactions with individuals beyond their close social network occur less frequently, approximately 1–3 days a month. Each social capital indicator exhibited significant differences (*p* < 0.001, analysis of variance) across the studied areas. Greater Manchester residents showed the highest level of network bridging (8.3), but lowest levels of social trust (4.0) and reciprocity (4.7). Utrecht residents showed the highest levels of trust (4.7), cooperativeness (4.5), and reciprocity (5.2), whilst Malmö residents led in network bonding (16.1) but they had the lowest network bridging (7.0) and cooperativeness (4.1).

The average score for perceived overall accessibility was 35.9 (S.E. = 6.5; median = 36), suggesting a generally satisfactory level of perceived accessibility, given the neutral point is 27. Each statement used to measure perceived overall accessibility was evaluated above the neutral point (i.e., 3). The statement ‘I can easily reach the supermarket or local shopping areas within my ideal travel time’ received the highest score at 4.2. Conversely, the statement ‘I do not have to spend more money on necessary travel in a week than I can afford’ received the lowest score at 3.6. Significant (*p* < 0.01, analysis of variance) across location differences in perceived overall accessibility were observed: Malmö residents reported the highest level of perceived overall accessibility (mean = 38.2), outperforming those in Utrecht (33.4) and Greater Manchester (33.4).

### Perceived overall accessibility and social capital

Regression results showed significant associations between perceived overall accessibility and social capital (Table 2). We found that a higher level of perceived accessibility was associated with higher levels of social trust (Coef. = 0.026; *p* < 0.001), cooperativeness (Coef. = 0.016; *p* < 0.01), reciprocity (Coef. = 0.047; *p* < 0.001), and network bonding (Coef. = 0.047; *p* < 0.001), after the data were controlled for individuals’ demographic and socioeconomic characteristics, travel patterns, and postcodes. There was no statistical relation between perceived overall accessibility and network bridging capital at the level of *p* < 0.10.

**Table 2:** The association between perceived accessibility and social capital

	Social Trust			Cooperativeness			Reciprocity			Network Bonding			Network Bridging		
	Coef	SE	Elasticity	Coef	SE	Elasticity	Coef	SE	Elasticity	Coef	SE	Elasticity	Coef	SE	Elasticity
Perceived overall Accessibility	0.026***	0.006	0.221	0.016**	0.006	0.134	0.047***	0.006	0.330	0.047***	0.013	0.106	0.008	0.012	0.036
R-squared	0.242			0.215			0.233			0.283			0.325		

All models are controlled by individuals’ demographic and socioeconomic characteristics, travel patterns (see, Appendices A & B), usage frequency of shared bikes and e-scooters, and postcodes

^**^p < 0.01; ***p < 0.001

Table 3 displays the results of DirectLiNGAM and skewness-based DDA on the dominant directions of significant associations. To interpret the table, the first column shows dominant directions detected by DirectLiNGAM. The second to fourth columns report the results of skewness-based DDA. These three columns respectively denote the difference in the absolute value of skewness Δ*s* and corresponding CI between the target (perceived overall accessibility → social capital) and alternative (social capital → perceived overall accessibility) models concerning the outcome and residual, as well as the dominant directions detected according to the previous two columns. Significant negative values of Δ*s* for the predictor and residual distributions indicate that perceived overall accessibility is more likely to be the cause of social capital, whilst significant positive values of Δ*s* suggest the reverse direction.

**Table 3:** Dominant directions between perceived overall accessibility and social capital

DirectLiNGAM	DDA		
Dominant direction	Δ*s*: Outcome distribution (90% CI)	Δ*s*: Residual distribution (90% CI)	Dominant direction
POA → Social Trust	− 0.190 (− 0.281, − 0.096)	− 0.185 (− 0.281, − 0.091)	POA → Social trust
POA → Cooperativeness	− 0.278 (− 0.373, − 0.183)	− 0.269 (− 0.365, − 0.171)	POA → Cooperativeness
POA → Reciprocity	− 0.114 (− 0.220, − 0.011)	− 0.092 (− 0.198, 0.015)	POA → Reciprocity
Network bonding → POA	− 0.194 (− 0.287, − 0.098)	− 0.202 (− 0.300, − 0.105)	POA → Network bonding

The first column shows dominant directions detected by DirectLiNGAM. The second to fourth columns report the results of DDA. These three columns respectively denote the difference in the absolute value of skewness between the target (perceived overall accessibility → social capital) and alternative (social capital → perceived overall accessibility) models concerning the outcomes and residuals as well as dominant directions detected according to the previous two columns

Abbreviation: POA (perceived overall accessibility). Results of kurtosis-based DDA are provided in Table 1 in Supplementary Material

Results of DirectLiNGAM and skewness-based DDA are highly similar: a higher level of perceived overall accessibility may contribute to higher levels of social trust, cooperativeness, and reciprocity. Skewness-based DDA and DirectLiNGAM reported a contradicting dominant direction for the association between perceived overall accessibility and network bonding. When using kurtosis-based DDA, the absolute differences in kurtosis (Δ*κ*) on the outcome/residual distributions failed to reach a statistical significance at *p* < 0.10 in the associations of perceived overall accessibility with each of the following factors: social trust, reciprocity, and network bonding. Nevertheless, considering that DDA focuses on the normality of outcome and residual distributions, which are jointly reflected by skewness and kurtosis indicators, our results on the dominant direction detected by DirectLiNGAM and skewness-based DDA remain relatively robust. It is noteworthy that skewness- and kurtosis-based DDA identified opposing dominant directions in the association between perceived overall accessibility and cooperativeness, suggesting DDA’s inability to identify the dominant direction of this association for our data.

### Perceived overall accessibility and SMM use

The regression analysis showed a negative association between the level of perceived overall accessibility and the frequency of using shared bikes (Coef. = -0.010; *p* < 0.10) (Table 4), *ceteris paribus*. However, no significant association between perceived overall accessibility and shared e-scooter use frequency was observed across the entire sample. This mirrors results from the model focusing on only Greater Manchester and Malmö residents, where shared e-scooters are available. For the dominant direction, DirectLiNGAM and skewness-based DDA presented consistent results regarding the perceived overall accessibility-shared bike use association, suggesting that a lower level of perceived overall accessibility may contribute to more frequent use of shared bikes (Table 5). However, results of kurtosis-based DDA contradict those of skewness-based DDA, suggesting a reverse dominant direction (see, Table 2 in Supplementary Material). Given DDA’s inability to ascertain the dominant direction between perceived overall accessibility and shared bike use frequency for our dataset, we will (primarily) leverage the findings from DirectLiNGAM to further discuss the relationship between perceived overall accessibility and SMM use in Sect. “Discussions and conclusion”.

**Table 4:** The association between perceived accessibility and SMM use frequency

	Shared Bike Use Frequency			Shared E-scooter Use Frequency		
	Coef	SE	Elasticity	Coef	SE	Elasticity
Perceived overall Accessibility	− 0.010*	0.005	− 0.175	− 0.004	0.005	− 0.079
R-squared	0.538			0.468		

All models are controlled by individuals’ demographic and socioeconomic characteristics, travel patterns, and postcodes

^*^p < 0.10

**Table 5:** Dominant directions between perceived accessibility and shared bike use frequency

DirectLiNGAM	DDA		
Dominant Direction	Δ*s*: Outcome distribution (90% CI)	Δ*s*: Residual distribution (95% CI)	Dominant direction
POA → Shared bike use frequency	− 0.142 (− 0.266, − 0.022)	− 0.155 (− 0.276, − 0.034)	POA → Shared bike use frequency

The first column shows dominant directions detected by DirectLiNGAM. The second to fourth columns report the results of DDA. These three columns respectively denote the difference in the absolute value of skewness between the target (perceived overall accessibility → shared bike use frequency) and alternative (shared bike use frequency → perceived overall accessibility) models concerning the outcomes and residuals as well as dominant directions detected according to the previous two columns. Results of kurtosis-based DDA are provided in Table 2 in Supplementary Material

*POA* Perceived overall accessibility

### SMM use and social capital

Multivariate analyses showed that SMM use was closely associated with social capital (Table 6). More frequent use of shared bikes was associated with higher levels of social trust (Coef. = 0.077; *p* < 0.01) and cooperativeness, (Coef. = 0.056; *p* < 0.05). However, there was no statistical relation between shared bike use frequency and reciprocity, network bonding, or network bridging at *p* < 0.10. Using the entire sample, we found that a higher frequency of using shared e-scooters was associated with a higher level of network bonding (Coef. = 0.106; *p* < 0.10), but the frequency of using shared e-scooters was not significantly associated with the level of social trust, cooperativeness, reciprocity, and network bridging. The results on shared e-scooter use-social capital relation remained highly similar regarding the coefficient and significance after we used Greater Manchester- and Malmö-only samples.

**Table 6:** The association between SMM use and social capital

	Social trust			Cooperativeness			Reciprocity			Network bonding			Network bridging		
	Coef	SE	Elasticity	Coef	SE	Elasticity	Coef	SE	Elasticity	Coef	SE	Elasticity	Coef	SE	Elasticity
Shared bikeuse frequency	0.077**	0.027	0.035	0.056*	0.026	0.026	0.020	0.026	0.008	− 0.058	0.059	− 0.007	0.067	0.055	0.017
Shared E-scooter use frequency	− 0.027	0.030	− 0.011	− 0.005	0.029	− 0.002	0.014	0.029	0..005	0.106*	0.064	0.012	0.090	0.060	0.021
R-squared	0.242			0.215			0.233			0.283			0.325		

All models are controlled by individuals’ demographic and socioeconomic characteristics, travel patterns (see, Appendices A & B), perceived overall accessibility, and postcode

^*^p < 0.10; *p < 0.05; **p < 0.01

For the dominant direction for the association between shared bike use and social capital, DirectLiNGAM and skewness-/kurtosis-based DDA suggested more frequent use of shared bikes might be an antecedent to higher levels of social trust and cooperativeness (Table 7 and Supplementary Material’s Table 3). For the association between shared e-scooter usage and social capital, both DirectLiNGAM and DDA indicated that a higher frequency of shared e-scooter use tended to be an antecedent to higher-level network bonding. In summary, our findings suggest that increased use of SMM may contribute to increased social capital.

**Table 7:** Dominant directions between perceived accessibility and social capital

DirectLiNGAM	DDA		
Dominant direction	Δ*s*: Outcome distribution (90% CI)	Δ*s*: Residual distribution (90% CI)	Dominant direction
Shared bike use frequency → Social trust	− 0.070 (− 0.183, 0.047)	− 0.055 (− 0.169, 0.060)	Shared bike use frequency → Social trust
Shared bike use frequency → Cooperativeness	− 0.121 (− 0.242, 0.000)	− 0.129 (− 0.249, − 0.009)	Shared bike use frequency → Cooperativeness
Shared E-scooter use frequency → Network bonding	− 0.496 (− 0.610, − 0.380)	− 0.494 (− 0.609, − 0.381)	Shared E-scooter use frequency → Network bonding

The first column shows dominant directions detected by DirectLiNGAM. The second to fourth columns report the results of DDA. These three columns respectively denote the difference in the absolute value of skewness between the target (SMM use frequency → social capital) and alternative (social capital → SMM use frequency) models concerning the outcomes and residuals as well as dominant directions detected according to the previous two columns. Results of kurtosis-based DDA are provided in Table 3 in Supplementary Material

## Discussions and conclusion

Our research investigated interrelationships between the use of SMM, perceived overall accessibility and social capital based on a questionnaire survey in three European countries. We find significant associations between SMM, perceived accessibility and social capital with variations between shared bikes and shared e-scooters. This is one of the first studies that investigates how social capital is linked to SMM. These findings have implications for transport research and planning, which we discuss in this section alongside considerations of the limitations of our findings and questions for future investigation.

### Substantive empirical findings

We explored three associations: associations (1) between the level of perceived overall accessibility and social capital; (2) between perceived overall accessibility and SMM use; and (3) between SMM use and social capital.

#### Perceived overall accessibility and social capital

We find that individuals with higher levels of perceived overall accessibility reported higher levels of social capital, encompassing social trust, cooperativeness, and reciprocity. While our findings are in line with existing studies that use spatial metrics as proxies for accessibility (Noland et al. 2016; Kamruzzaman et al. 2014; Utsunomiya 2016), our research extends beyond mere correlation to suggest directional relationships. Our results indicate that the dominant direction may flow from perceived overall accessibility to certain types of social capital. This direction of likely causation was mostly identified by both DirectLiNGAM and DDA, which increases confidence in the finding. Our research therefore lends empirical support to the potentially significant role of perceived overall accessibility in enhancing social capital.

#### Perceived overall accessibility and SMM use

Our results reveal complexity in associations between perceived overall accessibility and the use of SMM. Lower levels of perceived overall accessibility are associated with a higher frequency of using shared bikes, yet no significant association is found for the use of shared e-scooters. Our examination of the dominant direction indicates that a lower level of perceived overall accessibility may contribute to increased usage of shared bikes. This suggests that the perception of accessibility disadvantages in daily life tends to encourage the adoption of emerging urban mobility solutions, shared bikes in our case. Although this direction is only supported by DirectLiNGAM due to the incapability of DDA, its validity could also be corroborated by conceptual arguments. As we previously discussed, this may be attributable to the mechanisms of enacting adaptive behaviours, i.e., behaviours encompassing learned strategies that individuals employ to effectively navigate environmental challenges (Zheng et al. 2021). Evidence shows that by perceiving barriers posed by the environment, individuals may tend to learn new skills and/or adopt new measures to cope with the environment to reduce negative perceptions (Musterd et al. 2016). Additionally, individuals may not be inclined to frequently use a transport service that not only reduces their perceived daily accessibility but also incurs costs.

Previous studies suggest that increased SMM use tends to enhance individuals’ daily travel accessibility (Wang et al. 2022, Liu and Miller 2022, Smith and Schwieterman 2018, Qian and Niemeier 2019, Buehler and Hamre 2014, Filipe Teixeira et al. 2022). However, our findings suggest that this relationship is more nuanced. While lower perceived accessibility may encourage the use of shared bikes, it is not clear that this in turn increases perceived accessibility. Whether or not it does, is a question for further research. If further evidence confirms that shared bike use does contribute to improving perceived overall accessibility, then this could have significant policy implications for the development of more efficient, sustainable transport systems. If, however, it is found that shared bike use does not improve perceived accessibility, it would be of concern to planners, and be important to understand why. Potential explanations could be that while shared bike services have enough value to motivate people to use them, they are not considered to have sufficient availability or coverage to improve individuals’ perceptions of accessibility.

The fact that there is no significant association between perceived accessibility and e-scooters is quite striking, and a challenge to prospects for using shared e-scooters to improve accessibility in urban mobility. Nevertheless, this lack of association could have multiple explanations. The coverage of shared e-scooter services was limited in Greater Manchester, and absent in Utrecht (and thus not included), which may have led to a low effect size compared to the variation. Moreover, business models used to provide shared e-scooters and shared bikes differ, as does the cost of use. For example, in Greater Manchester, each ride of shared bikes costs 50 pence to unlock and 5 pence per minute to ride, whilst a shared e-scooter entails a £1 unlocking fee and a 15-pence per minute charge. This may lead to differences in the affordability and geographical coverage of these two types of SMM. Finally, whereas shared bikes are mostly used for transport, it might be the case that the use of e-scooters is more recreational, reducing the link with perceived accessibility at the current moment (Hardt and Bogenberger 2019). Each of these explanations may be explored in future research.

#### SMM use and social capital

Our findings suggest that increased use of shared bikes may contribute to higher levels of social trust and cooperativeness, while greater use of shared e-scooters may contribute to enhanced network bonding. This suggests that SMM potentially offers benefits that are up to now perhaps insufficiently acknowledged. As we previously discussed, individuals who perceived greater accessibility disadvantages were more inclined to use shared bikes. Synthesising these findings, two implications emerge: (1) SMM may indirectly foster greater well-being and social inclusion by augmenting social capital amongst users, and (2) shared bikes may reduce differences in social capital stemming from interpersonal disparities in perceived accessibility. However, it would be premature to conclude unequivocally that the expansion of SMM will benefit social capital building over a wider population, encompassing non-users. For example, SMM can also be linked to decreased accessibility, in terms of hindering pedestrians and creating conflicts between road users (i.e. physical limitations) (James et al. 2019; Duran-Rodas et al. 2020). Our analyses focused on the impact of the use of SMM on social capital, but there may be negative impacts of the existence/expansion of SMM on non-users. Further analyses could focus on the impacts of other aspects of SMM on perceived accessibility and social capital, such as the proximity of stations, the number of vehicles in the area, and the levels of use in the area, to extend the scope to non-users.

Our findings and the further questions raised can contribute to resolving the increasingly tense debates on SMM presence in cities (Aman and Smith-Colin 2021; An et al. 2023). With the rapid expansion of SMM services and a significant increase in user numbers in recent years, policy and media discussions in cities such as Paris (The Guardian 2023; Lipovsky 2021), Dallas (Aman and Smith-Colin 2021), and Malmö (Voi 2023) have increasingly centred around imposing restrictions in recent years, and in some cases, complete bans on certain types of SMM, particularly shared e-scooters. While it is understandable given the hindrance SMM could cause in some places, policymakers should not ignore the positive aspects that these forms of mobility could offer to their inhabitants. Striking a balance between access to vehicles to stimulate the use of SMM by inhabitants, while managing the growth, street presence, and the behaviour of users is thus likely the best way forward.

### Investigating likely causal direction

The multitude of challenges inherent in data collection, such as exorbitant costs, significant time expenditure, and participant attrition, coupled with the impracticability of experimental or natural transport interventions (e.g., randomly assigning a population subset to use SMM), necessitates the predominance of cross-sectional non-intervention designs in transport-related empirical research. Without sophisticated statistical techniques, a key limitation of such designs rests in their inability to ascertain the directionality between the variables under investigation. This can lead to ambiguous findings, which may, in turn, compromise the applicability and impact of the research outcomes. We suggest that the applied causal discovery methodologies–DirectLiNGAM and DDA–hold the potential for illuminating the (likely) causal relationship between transport and social capital, in addition to other transport-related subjects. As demonstrated in our study, these causal discovery approaches (among others) provide a potentially feasible strategy to explore the dominant direction between variables in a non-interventional setting.

However, we underscore that these two approaches (and other similar ones, see, Spirtes and Zhang (2016) for the review) must be used with caution. First, we recommend the use of multiple causal discovery approaches in research, especially those built upon different statistical theories, to ensure the robustness of the findings. In our research, for example, we found discrepancies in the dominant direction of associations identified using DirectLiNGAM and DDA, such as between network bonding and perceived overall accessibility. This suggests that reliance on a single method could yield erroneous directional inferences. Second, we assert the importance of a theoretically informed application of these approaches. That is, causal discovery approaches should function as supportive instruments for the preliminary verification of hypotheses rather than data-driven explorations. This arises from the fact that these methods, being more or less sensitive to factors such as data quality and the satisfaction of statistical assumptions, can make achieving unbiased results challenging. Consequently, a lack of theoretical underpinning for the inferred dominant directions could lead to unreliable conclusions, and diminish the interpretability of the findings. Third, researchers must not neglect the statistical assumptions embedded within these approaches. For example, most causal discovery approaches, including DirectLiNGAM and DDA, operate under the assumption of the absence of common hidden confounders in the data. Despite being a relatively strong statistical assumption, which is often difficult to entirely satisfy, researchers should meticulously scrutinise control variables to fulfil this assumption to the greatest extent possible.

### Limitations and future directions

Our research used innovative methods in the field of transport to investigate the likely causal relationships between the use of SMM, perceived overall accessibility and social capital based on data collected in three countries. However, our research has several limitations. First, and foremost, while the applied methods facilitate potential causal inferences, our data are cross-sectional. Therefore, we cannot draw ‘real’ causal conclusions. Future research could strengthen causal claims by employing longitudinal data and leveraging more robust designs such as natural experiments.

Second, the data are self-reported, and consequently misreporting either deliberate or accidental cannot be ruled out. The reported frequency of SMM use is particularly vulnerable to this limitation. Subsequent research could enhance measurement accuracy by utilising GPS-based trajectory data to quantify SMM usage intensity.

Third, while our recruitment strategy targeted the general population, issues with data representativeness may restrict the generalisability of our findings to specific subpopulations and areas, and limit their relevance to real-world contexts. For example, we find that individuals may use shared bikes more frequently to compensate for perceived accessibility disadvantages. However, since younger adults aged 18 to 34 is overrepresented in our data, it is inconclusive whether this behaviour extends to other age groups, especially older adults, who face different physical challenges to use shared bikes. Our findings also suggest that increased use of SMM may be an antecedent to increased social capital, but evidence shows that access to and acquisition of social capital vary across different genders, income levels, and educational backgrounds (Schiff 2010; Otero et al. 2023; Burt 1998; Lin 2000; Huang et al. 2009). Given the underrepresentation of certain income groups, males, and individuals without higher education qualifications in our data, caution is advised in applying our conclusions broadly. Future research should employ more representative data or focus on specific subpopulations to better understand these relationships and enhance the generalisability of findings.

Fourth, the multinational scope of our dataset broadens the geographical reach of our research. Nonetheless, our research did not extend to the specific geographical variations in the interrelationships between perceived overall accessibility, SMM use, and social capital. Various factors, such as the asynchronous introduction of SMM, disparities in geographical coverage, and differences in the availability of other transport modes, could potentially affect the use of SMM and its implications. Despite these considerations, the methodological control for the area of residence in our estimations ensures that such geographical differences should not largely impact our core findings. Nevertheless, a nuanced examination of inter-country differences could provide actionable insights for location-specific SMM development strategies.

## Electronic supplementary material

Below is the link to the electronic supplementary material.

Supplementary file1 (DOCX 16 kb)

## Appendix A

See Table

**Table 8:** Summary of individuals’ demographic and socioeconomic characteristics

Variables	Greater Manchester (Share) (%)	Utrecht (Share) (%)	Malmö (Share) (%)	Overall (Share) (%)
*Gender*				
Male	44.8	44.1	43.5	43.9
Female	53.6	55.2	56.1	55.3
Other	1.6	0.7	0.5	0.8
*Age*				
18–24	15.2	23.7	7.0	13.2
25–34	25.8	30.0	28.7	28.4
35–44	19.3	22.8	19.7	20.4
45–54	14.6	12.2	13.4	13.3
55–64	15.7	7.8	15.4	13.5
> 65	6.3	3.5	15.4	10.3
Prefer not to say	3.1	0.0	0.4	0.9
*Country of origin*				
The studied country	87.9	87.0	86.4	86.9
Other	12.1	13.0	13.6	13.1
*Living alone*				
Yes	30.7	14.1	26.3	24.0
No	69.3	85.9	73.7	76.0
*Presence of children in the household*				
Yes	30.3	42.2	29.1	32.8
No	69.7	57.8	70.9	67.2
*Employment status*				
Working full-time	55.8	67.2	61.8	61.9
Working part-time	17.5	6.7	3.3	7.2
Unemployed	3.4	4.6	4.5	4.3
Homemaker	3.1	6.1	2.4	3.6
Student	5.2	8.0	9.1	7.9
Retired	5.8	3.3	16.2	10.6
Unable to work	7.6	3.9	2.1	3.7
Other	1.6	0.2	0.7	0.8
*Household income*				
Q1 (Low)	16.4	18.9	6.3	11.8
Q2	18.8	19.4	10.8	14.8
Q3	20.2	18.9	21.3	20.4
Q4	17.0	17.0	20.2	18.7
Q5 (High)	13.0	15.7	24.7	19.8
Prefer not to say	14.6	10.0	16.7	14.5
*Educational attainment*				
Higher education	79.6	49.4	52.4	57.5
Upper secondary	8.7	24.4	38.3	28.3
Secondary	7.6	21.7	0.0	7.3
Other	4.0	4.4	9.3	6.9
*Long-term health condition*				
Yes	23.8	20.4	17.7	19.7
No	57.8	66.5	74.2	68.6
Prefer not to say	18.4	13.1	8.1	11.7

^8^.

## Appendix B

See Table

**Table 9:** Summary of individuals’ access to transport options and travel patterns

Variables	Greater manchester (Share) (%)	Utrecht (Share) (%)	Malmö (Share) (%)	Overall (Share) (%)
*Access to cars*				
Sole access	37.2	48.5	34.6	38.8
Shared access	45.1	25.7	43.1	39.0
No regular access	2.5	23.7	18.8	16.5
Other	15.2	2.0	3.6	5.7
*Access to bikes*				
Sole access	66.4	37.0	78.0	64.7
Shared access	12.3	14.1	4.7	8.8
No regular access	21.1	48.9	16.7	26.1
Other	0.2	0.0	0.6	0.3
*Ownership of other micromobility*				
Yes	42.6	21.3	24.7	27.7
No	57.4	78.7	75.3	72.3
*Walking frequency*				
Almost never	4.7	4.3	2.2	3.3
1–5 days a year	1.8	0.7	0.8	1.0
6–11 days a year	4.5	2.2	0.8	2.0
1–3 days a month	6.1	3.7	4.3	4.5
1–3 days a week	11.4	12.6	10.3	11.1
4–6 days a week	17.3	11.7	12.1	13.1
Daily	54.3	64.8	69.4	64.9
*Car use frequency*				
Almost never	13.2	11.9	14.9	13.7
1–5 days a year	3.1	2.0	3.6	3.1
6–11 days a year	4.3	3.9	6.3	5.2
1–3 days a month	16.8	7.2	16.2	14.0
1–3 days a week	27.8	22.4	25.9	25.4
4–6 days a week	14.3	15.0	15.9	15.3
Daily	20.2	37.6	17.3	23.2
*Local public transport use frequency*				
Almost never	19.5	16.1	14.4	16.0
1–5 days a year	15.0	9.6	13.3	12.7
6–11 days a year	11.0	8.9	15.7	12.9
1–3 days a month	17.5	23.0	22.1	21.4
1–3 days a week	21.1	21.1	18.2	19.6
4–6 days a week	11.0	10.4	9.5	10.1
Daily	4.9	10.9	6.7	7.4
*Regional public transport use frequency*				
Almost never	16.1	23.1	15.2	17.5
1–5 days a year	15.5	16.3	19.3	17.7
6–11 days a year	10.8	10.9	18.7	14.9
1–3 days a month	21.1	26.7	22.7	23.4
1–3 days a week	19.5	11.5	10.3	12.6
4–6 days a week	11.4	7.0	8.6	8.8
Daily	5.6	4.4	5.2	5.1
*Personal bike use frequency*				
Almost never	20.2	46.3	22.4	28.2
1–5 days a year	4.0	4.3	7.1	5.7
6–11 days a year	4.0	6.9	7.5	6.6
1–3 days a month	9.4	14.4	12.1	12.1
1–3 days a week	14.1	12.4	12.9	13.0
4–6 days a week	18.8	7.8	13.9	13.4
Daily	29.1	8.0	24.1	21.0
*Personal e-bike use frequency*				
Almost never	56.5	65.7	75.5	68.8
1–5 days a year	5.2	6.5	4.3	5.1
6–11 days a year	3.6	5.9	2.7	3.7
1–3 days a month	8.1	6.7	4.1	5.6
1–3 days a week	11.2	7.8	3.8	6.5
4–6 days a week	5.4	4.3	3.9	4.3
Daily	10.1	3.1	5.6	5.9

^9^.

## Footnotes

1 In 2021, the gender distribution was 49% male and 51% female for both Greater Manchester and Utrecht, compared to 45% versus 54% and 44% versus 56% in our data, respectively; Malmö reported a gender distribution of 48% male and 52% female (44% versus 56% for our data). In 2023, the percentage of native-born individuals was 83% in Greater Manchester, 86% in Utrecht, and 64% in Malmö, with our data showing 88%, 87%, and 86%, respectively.

2 In 2022, individuals aged 17–34 comprised 29% of the population aged 17 and over in Greater Manchester, and 27% in Utrecht (41% and 54% for our data). For Malmö, individuals aged 65 and over accounted for 19% of the population aged 17 and over (10% for our data).

3 According to the national statistics reported by OECD (2024), in 2022, 58%, 56%, and 53% of individuals in the UK, the Netherlands, and Sweden respectively possessed higher education qualifications. In our data, the corresponding figures were 80%, 49%, and 52% respectively.
