# Supplementary material for: Shared micromobility, perceived accessibility, and social capital
Source: Transportation (Amst). 2024 Aug 21;53(2):1025–60. doi: 10.1007/s11116-024-10521-5 (PMC12968100; doi:10.1007/s11116-024-10521-5)
Supplement: Supplementary file 1 — Supplementary file1 (DOCX 16 kb) [file 11116_2024_10521_MOESM1_ESM.docx]

**Table 1** Kurtosis-based DDA on the association between perceived overall accessibility and social capital.

| DDA | | |
| --- | --- | --- |
| Δ*k*: Outcome Distribution (90% CI) | Δ*k*: Residual Distribution (90% CI) | Dominant Direction |
| 0.187 (-0.009, 0.300) | 0.149 (-0.051, 0.267) | Undetermined (Social Trust & POA) |
| 0.345 (0.144, 0.421) | 0.301 (0.110, 0.396) | Cooperativeness → POA |
| 0.057 (-0.125, 0.205) | 0.079 (-0.116, 0.240) | Undetermined (Reciprocity & POA) |
| 0.009 (-0.132, 0.117) | 0.035 (-0.117, 0.124) | Undetermined (Network Bonding & POA) |

**Table 2** Kurtosis-based DDA on the association between perceived overall accessibility and SMM use.

| DDA | | |
| --- | --- | --- |
| Δ*k*: Outcome Distribution (90% CI) | Δ*k*: Residual Distribution (90% CI) | Dominant Direction |
| 0.881 (0.601, 1.044) | 0.835 (0.566, 0.999) | Shared Bike Use Frequency → POA |

**Table 3** Kurtosis-based DDA on the association between SMM use and social capital.

| DDA | | |
| --- | --- | --- |
| Δ*k*: Outcome Distribution (90% CI) | Δ*k*: Residual Distribution (90% CI) | Dominant Direction |
| -0.639 (-0.872, -0.408) | -0.616 (-0.854, -0.381) | Shared Bike Use Frequency → Social Trust |
| -0.571 (-0.801, -0.344) | -0.555 (-0.786, -0.331) | Shared Bike Use Frequency → Cooperativeness |
| -1.264 (-1.467, -0.952) | -1.239 (-1.447, -0.929) | Shared E-scooter Use Frequency → Network Bonding |
